# Accounting for uncertainty in model-based prevalence estimation: paratuberculosis control in dairy herds

**DOI:** 10.1186/1746-6148-8-159

**Published:** 2012-09-10

**Authors:** Ross S Davidson, Iain J McKendrick, Joanna C Wood, Glenn Marion, Alistair Greig, Karen Stevenson, Michael Sharp, Michael R Hutchings

**Affiliations:** 1 Disease Systems, SAC, , West Mains Road, Edinburgh EH9 3JG, UK; 2, Biomathematics & Statistics Scotland, JCMB, The King’s Buildings, Edinburgh EH9 3JZ, UK; 3, Moredun Research Institute, Bush Loan, Penicuik EH26 0PZ, UK; 4, Veterinary Laboratories Agency, Lasswade, Bush Loan, Penicuik EH26 0PZ, UK

## Abstract

**Background:**

A common approach to the application of epidemiological models is to determine a single (point estimate) parameterisation using the information available in the literature. However, in many cases there is considerable uncertainty about parameter values, reflecting both the incomplete nature of current knowledge and natural variation, for example between farms. Furthermore model outcomes may be highly sensitive to different parameter values. Paratuberculosis is an infection for which many of the key parameter values are poorly understood and highly variable, and for such infections there is a need to develop and apply statistical techniques which make maximal use of available data.

**Results:**

A technique based on Latin hypercube sampling combined with a novel reweighting method was developed which enables parameter uncertainty and variability to be incorporated into a model-based framework for estimation of prevalence. The method was evaluated by applying it to a simulation of paratuberculosis in dairy herds which combines a continuous time stochastic algorithm with model features such as within herd variability in disease development and shedding, which have not been previously explored in paratuberculosis models. Generated sample parameter combinations were assigned a weight, determined by quantifying the model’s resultant ability to reproduce prevalence data. Once these weights are generated the model can be used to evaluate other scenarios such as control options. To illustrate the utility of this approach these reweighted model outputs were used to compare standard test and cull control strategies both individually and in combination with simple husbandry practices that aim to reduce infection rates.

**Conclusions:**

The technique developed has been shown to be applicable to a complex model incorporating realistic control options. For models where parameters are not well known or subject to significant variability, the reweighting scheme allowed estimated distributions of parameter values to be combined with additional sources of information, such as that available from prevalence distributions, resulting in outputs which implicitly handle variation and uncertainty. This methodology allows for more robust predictions from modelling approaches by allowing for parameter uncertainty and combining different sources of information, and is thus expected to be useful in application to a large number of disease systems.

## Background

Simulation studies provide a useful insight into the dynamics of epidemiological systems and can be used to obtain a variety of predictions, such as the relative gains expected from different types of control. Difficulties often arise in the parameterisation of such models, as results may depend critically on parameters whose values are poorly understood, or which vary greatly between individual populations. A lack of knowledge of the value of a parameter is referred to here as uncertainty, and may represent either a lack of appropriate experimental studies or limitations in current methods for measurement. Even if it were possible to know the value of each parameter in any given location or population, there may be variability in their values between locations or populations, as well as variability arising from inherent unpredictability in the system even for fixed parameters (stochasticity). An example of an infection exhibiting a high degree of uncertainty and variability in parameter values is that of paratuberculosis, or Johne’s disease, a chronic inflammation of the intestine caused by the presence of *Mycobacterium avium* subsp. *paratuberculosis* (Map).

The low sensitivity of diagnostic tests and problems associated with culturing Map bacteria and with carrying out experimental infections make laboratory studies difficult. Furthermore the disease has a long and variable incubation period of many months or even years and generally clinical cases within a given herd occur only sporadically; indeed many infected animals may never be recognized as such. The most important source of infection is the faeces of infected animals [1], so differing environmental conditions combined with the long incubation times are likely to contribute to a high degree of variability between farms. For example, the survival of Map in pasture, or the relationship between levels of bacterial contamination and the animal-level force of infection, are likely to vary greatly on different farms, depending on factors such as soil acidity, micro-climate and details of the management practices of the farm. It is essential that such uncertainty and parameter variability are properly accounted for when evaluating potential control options.

We have developed an individual based model which incorporates key aspects of the stochastic dynamics of paratuberculosis transmission in combination with a detailed description of the management practices applicable to a typical Scottish dairy herd. Stochastic individual based modelling techniques have been applied with considerable success to epidemiological systems [2]. Stochastic models can show considerable differences from their mean field equivalents [3], particularly in cases with a high degree of nonlinearity (when fluctuations can give rise to divergences from deterministic models), cases in which it is required to have a model of the inherent variability in the system, or situations with low prevalence where the statistics of disease extinction are of interest [4]. The stochastic nature of the model described here accounts for within-farm variability in the processes of disease transmission, progression and detection, and provides a suitable example with which to illustrate the use of the statistical methodology which we have developed to compare the impacts of control options.

In the approach taken here some of the parameters whose values are considered to be the most poorly known or to be the most variable between farms were assigned independent distributions of values. Parameters which have been treated in this manner shall be referred to as *sampled* parameters. We used independent distributions because the literature typically does not provide information on the correlations between parameters. Latin Hypercube Sampling (LHS) is an efficient means of sampling and combining parameters to generate a collection of *K* sample parameter sets, which gives coverage of the entire distribution for each parameter (see e.g. [5]). This is achieved by dividing each distribution into *K* equally probable intervals, which can then be sampled and combined into the parameter sets for running the model using a Latin hypercube design. A recent review [6], highlighting a number of important features which have yet to be explored in paratuberculosis modelling research emphasised treatment of parameter uncertainty and model variability as key areas for improvement. Previous attempts to model the epidemiology of paratuberculosis in cattle have ranged from the largely theoretical (e.g. Collins and Morgan, 1991 [7], where parameterisation was not considered in depth), to more detailed exercises which used published literature and expert opinion to parameterise models [8-11], although a more recent model has carried out a more detailed sensitivity analysis [12,13].

In order to account for correlations between parameters and to incorporate additional information available from observed prevalence data, a statistical methodology was developed which assigns weights to the sampled parameter sets by quantifying the model’s resultant ability to reproduce the prevalence data. Outputs were subsequently obtained by running the model for all sampled parameter vectors with the outputs renormalized according to the assigned weights. Once the parameterisation and weightings are in place the model can be used to investigate the impact of control strategies on the infection. This involves modelling the test procedure and applying it to individual model realisations, prior to the subsequent reweighting of the results. The reweighting was carried out using the weights obtained in the ‘reference scenario’ in which the only control strategy in place was the removal of clinical animals, in order to reflect the conditions under which the data were collected. In this way we have been able to produce probabilistic assessments of different control strategies that combine model assumptions, uncertain information about parameter values found in the literature and limited data on within-herd prevalence.

In section “Paratuberculosis model design” we describe the model of paratuberculosis infection dynamics that has been designed for this study, and present its behaviour for a fixed set of parameters. The test models and infection management measures which were evaluated are also described. In section “Latin hypercube sampling and reweighting parameter sets” we go on to describe in detail how the parameter uncertainty has been factored into the model by the use of the reweighted Latin hypercube sampling scheme. In section “Results and discussion” the results before and after the reweighting process are compared, showing how the reweighted results provide a close reproduction of the prevalence data upon which the weighting is based. We then examine the impact of test and cull control strategies, both with and without husbandry measures in place to control the spread of infection between individuals.

## Methods

### Paratuberculosis model design

The key aspects of paratuberculosis infection that we intended to capture in our model were the impact of herd management practices, the spread of infection through the environment, vertical and pseudo-vertical transmission from dam to calf, and the influence of imperfect tests on the effectiveness of control strategies.

A continuous time stochastic model was developed by assigning mean rates to the various possible events, from which inter-event times can be generated using the Gillespie algorithm [2]. Although the infection model is in continuous time, management decisions are taken on a discrete time basis, with a time step of one week, in order to reflect typical farm routines. Individuals are assigned one of four management states (calf, heifer, pregnant and non-pregnant productive animals) as well as one of five infection states (susceptible, infected but not shedding, low shedding, high shedding and clinical). As the approach is computationally intensive, the model and reweighting procedure were constructed in the C programming language.

#### Herd Management

The herd was assumed to be closed (new animals are not introduced to the farm). Control methods which are found to be appropriate in the closed case will still be useful in the open herd situation if coupled with sensible purchasing strategies to minimize the risk of importing infection into the herd. The converse is not true, since the long-term epidemiological state of an open herd will be critically driven by the level of infection imported into the herd. Therefore we have considered the long-term behaviour of the epidemic following the introduction of a single infected animal onto the farm. For the purposes of the current study a herd size of 90 dairy cattle was used, a figure which reflects average herd sizes in Scotland circa 2000 [14], as well as the mean of 81 animals in the data set [15] used in the reweighting procedure, described in section “Latin hypercube sampling and reweighting parameter sets”. A spread rather than seasonal calving strategy was assumed, where individual births can occur at any point in the year so that no seasonal variation was included in the model. At birth, it was assumed that 50% of individuals are male, with these individuals being immediately culled. Furthermore 8% of female calves are born dead or abnormal and removed from the model. After a fixed time span of 6 months individuals are moved into the next management state, “heifer”, in order to form a supply of new animals for the milking herd. A calf mortality rate *μ*_*c*_ of 0.0085 per animal per month was assumed, corresponding to an overall calf mortality of 5% over 6 months, as given by the Milk Development Council [16].

The herd was assumed to be operating under a set stocking management practice, in which a constant number of productive animals is maintained. Animals in the heifer class are matured until they reach an age of 16 months. During this period they have an instantaneous mortality rate *μ*_*h*_ of 0.0011 per animal per month [17]. In order to keep the spread calving pattern intact, the death of an individual in the milking herd will result in the selection of a replacement from those heifers in the age range of 14 to 16 months, with a view to calving occurring at around 24 months. Heifers which have not been selected by the time they reach 16 months are culled [17,18], while if a replacement is needed when the pool of 14 to 16 month old animals is empty, a clean animal is bought in.

Productive animals are pregnant for a period of 9 months, and then left empty for 4 months [18]. In order to calculate mortality rates for productive animals an estimate for the annual replacement fraction in a typical Scottish dairy herd of 0.242 was used, based on the Scottish Government Economic Report on Scottish Agriculture [14]. The proportion of culls which are due to age related factors was taken from literature estimates of 0.11 [18] and 0.05 [19]. Combining these figures gave estimates of the fraction of the herd culled annually for reasons not related to age, *q*, of 0.216 and 0.230 respectively. As this range is fairly narrow we take a fixed intermediate value of *q*=0.22. This proportion is further partitioned into a fraction due to reproductive failure, taken as 0.292 [19], with the remaining 70.8*%*being assigned to other non-age related causes. An exponential inter-event time distribution was assumed, giving an instantaneous mortality rate for non-pregnant animals *μ*_*a*_=−ln(1−0.708*q*) per year. As animals are pregnant for 9 months followed by 4 months empty, the expected length of pregnancy in a randomly selected 12 month block is 108/13. Using this to generate the excess mortality *μ*_*p*_associated with pregnancy gives a total mean monthly rate for pregnant animals of

(1) $$\mu_{a}+\mu_{p}=-\ln(1-0.708q)/12-13\ln(1-0.292q)/108.$$

An additional source of mortality in productive animals is culling due to decline in milk yield. In this model it was assumed that individuals are kept for a fixed number of lactations, *n*, and culled upon reaching this limit. The value of *n* is fixed for a particular farm or, equivalently, sample parameter set, but due to the wide range of values quoted in the literature was treated as one of the sampled parameters, with values between 5 and 12 lactations.

#### Infection model

Due to the long incubation period of paratuberculosis, it was assumed that no calves are in the shedding states of the model [20]. The principal route of transmission applicable to all animals is by ingestion of bacteria from the environment. In addition to the average level of environmental bacteria, adult (heifer and productive) animals are exposed to a further bacterial source term representing an increased level around a clinical individual, whereas it was assumed that calves have little direct contact with animals other than their dam. Let *c*(*t*) be the level of bacteria in the environment at time *t* and *Z*_*a*_(*t*), *Y*_*ah*_(*t*) and *Y*_*al*_(*t*) be the number of clinical, high and low shedding adults respectively. The number of sub-clinical individuals is denoted by *X*_*a*_(*t*) for adults and *X*_*c*_(*t*) for calves.

Adult animals become infected at a rate

(2) $$\frac{\beta_{d}}{N_{a}}Z_{a}(t)+\frac{\beta_{\mathrm{ai}}}{N_{a}}f_{a}(c(t)),$$

where *β*_*d*_and *β*_*ai*_are parameters controlling the overall rate of direct and indirect infection, while *N*_*a*_ is the total number of adult individuals. The function *f*_*a*_(*c*(*t*)) defines the relationship between the level of bacteria in the environment and the force of infection on a susceptible adult. For calves, infection through the environment is modelled in a similar manner to that in adults, with a rate

(3) $$\frac{\beta_{\mathrm{ci}}}{N_{c}}f_{c}(c(t)),$$

where *β*_*ci*_is a further parameter controlling the rate of indirect infection of calves, and *f*_*c*_(*c*(*t*)) defines the relationship between the level of bacteria in the environment and the force of infection on a susceptible calf.

Where a calf is born to a shedding animal both *in utero* and *post partum* routes of infection were included. For *in utero* infection, the probability *p*_*iu*_of being infected at birth is given by

(4) $$\begin{array}{ll} p_{\mathrm{iu}}=\left\{\begin{array}{c} \frac{\log(\alpha_{\mathrm{low}})}{\log(\alpha_{\mathrm{high}})}p_{\text{max}}\;\text{for a low shedding animal}\\ p_{\text{max}}\;\text{for a high shedding animal} \end{array}\right) & \; \end{array}$$

in which *α*_*low*_ and *α*_*high*_ are shedding rates, defined below, and *p*_max_is a sampled parameter.

In order to represent *post partum* infection an additional term is added to the *in utero* probability, so that the total probability of vertical infection is given by

(5) $$p_{v}=p_{\mathrm{iu}}+\mathrm{u\phi}\left(1-p_{\mathrm{iu}}\right).$$

Here *u* is a sampled parameter taken from a *U*(0,1) distribution, representing the extent to which *post partum* infection through routes such as infected milk/colostrum is present in the model, while *ϕ*is a parameter which is used to represent infection management on the farm by limiting dam-calf contact, as will be discussed in section “Control measures”. In the absence of management interventions (*ϕ*=1) the probability of *post partum* infection will range from *p*_*iu*_ to 1. It was assumed that calves have little direct contact with animals other than their dam. Calf to calf transmission has recently been demonstrated [21], however we do not include this route as it is assumed to be a low probability event given the low levels of shedding.

The response functions *f*_*a*_(*c*) and *f*_*c*_(*c*) describe the infective impact of environmental bacteria. The true infective impact of any environmental infection is difficult to model as it will depend on the distribution of the bacteria and the grazing habits of the animals [22,23]. For this reason it is more useful to assume a simple functional form for this response, which will be nonlinear with a sigmoidal form obeying the natural constraints *f*(0)=0 and lim_*c*→*∞*_*f*(*c*)=*K*. The constant *K* can be set to 1 without loss of generality as its value can be absorbed into the values of *β*_*d*_, *β*_*ai*_ and *β*_*ci*_. Given these constraints a piecewise linear function was used, as this has relatively few parameters involved and will allow more effective sampling, hence

(6) $$f(c(t))=\left\{\begin{array}{c} 0\;\;\;\;\text{if}\;c<c^{\min}\\ \frac{c-c^{\min}}{c^{\max}-c^{\min}}\;\;\;\text{if}\;c^{\min}<c<c^{\max}\\ 1\;\;\;\;\text{if}\;c>c^{\max} \end{array}\right)$$

with different values $c_{c}^{\min}$, $c_{c}^{\max}$, $c_{a}^{\min}$ and $c_{a}^{\max}$ for calves and adults. Using a different function *f*_*c*_(*c*(*t*)) for calf infection from the environment allowed for calves to be modelled as being at a higher risk of infection for a given level of contamination. The models of [8-11] all assume that initial infection can only occur up to one year of age, whereas here it has merely been assumed that the rate of infection is higher for calves.

Once infected each animal is assigned a sub-clinical phase of duration *T*_*i*_, which is drawn from a Gamma distribution *Γ*(*ν*_*c*_*λ*_*c*_) for calves and *Γ*(*ν*_*a*_*λ*_*a*_) for adults. The parameters in these distributions were obtained by fitting to published data [24-26]. The sub-clinical period for each animal *i* is divided into the non-shedding phase of duration *f*_1_*T*_*i*_, the low shedding phase of duration *f*_2_*T*_*i*_ and the high shedding phase of duration (1−*f*_1_−*f*_2_)*T*_*i*_. These classes were designed to be analogous to those of [27], with disease states also being similar to those used in [10].

Animals in the low shedding phase excrete bacteria at a rate *α*_*low*_, while those in the high shedding state do so at a rate *α*_*high*_. In order to calculate these shedding parameters, an underlying exponential shedding process for an animal infected at time *t*_0_ of $\alpha (t-t_{0}-f_{1}T_{i})=Ae^{\lambda (t-t_{0}-f_{1}T_{i})}$was assumed. At the start of the low shedding phase, a shedding rate *α*(0)=*α*_*min*_ was assumed, which is derived using the faecal test model described in section “Control measures” assuming a mean detection probability for an animal in the low shedding state equal to 0.2, as given in [27]. The final shedding rate *α*((1−*f*_1_)*T*_*i*_)=*α*_*max*_, obtained when an animal reaches the clinical phase of infection, is likely to be the most critical to the epidemiology and was thus treated as a sampled parameter. Given these values it is straightforward to calculate *A* and *λ*. The values of *α*_*low*_and *α*_*high*_were then calculated as averages of the exponential process over the corresponding time periods,

(7) $$\alpha_{\mathrm{low}}=\frac{1}{f_{2}T_{i}}\int_{0}^{f_{2}T_{i}}\alpha (t-t_{0}-f_{1}T_{i}),$$

(8) $$\alpha_{\mathrm{high}}=\frac{1}{(1-f_{1}-f_{2})T_{i}}\int_{f_{2}T_{i}}^{(1-f_{1})T_{i}}\alpha (t-t_{0}-f_{1}T_{i}).$$

Explicitly modelling shedding in this manner, allowing for some individuals to shed at extremely high rates, incorporates some of the issues addressed by Mitchell et al. [11], although the current model only allows for variability in shedding rates between realisations, rather than explicit inclusion of a small number of animals shedding at an exceptionally high rate. It is likely that such animals would have a relatively limited impact on the long term dynamics of infection as they would be easily picked up by faecal tests [11], however they would contribute to additional variability in the observed dynamics, a feature which would not be observed in a deterministic model such as that of Mitchell et al.

Individuals in the clinical state were assigned a disease induced mortality *μ*_*i*_ which is added to their basic mortality rates, *μ*_*h*_, *μ*_*a*_ or *μ*_*a*_ + *μ*_*p*_, in order to reflect culling of such animals. It was assumed that an animal is culled one week after entering the clinical state, equivalent to a value for *μ*_*i*_of 2 per animal per month.

#### Environmental infection

Mitchell et al. do not model environmental infection, but suggest that doing so would be worthwhile. The level of environmental contamination *c*(*t*) was modelled as a deterministic process with a linear decay rate *δ*, which is a sampled parameter having a range of values that gives an infective dose a half life of between 28 and 133 days, consistent with data in [28]. The value of *c*(*t*) is governed by the differential equation

(9) $$\frac{\mathrm{dc}(t)}{\mathrm{dt}}=\alpha_{\mathrm{low}}Y_{\mathrm{al}}(t)+\alpha_{\mathrm{high}}Y_{\mathrm{ah}}(t)+\alpha_{\max}Z_{a}(t)-\mathrm{\delta c}(t).$$

A deterministic process was chosen as this quantity is likely to be very high, and hence the size of fluctuations will be relatively small. A stochastic process for a population of such a size would be extremely numerically intensive, whereas the deterministic process can be solved relatively straightforwardly. As *Y*_*al*_, *Y*_*ah*_ and *Z*_*a*_ are stochastic variables, the function *c*(*t*) will exhibit random changes in the growth rate at discrete time points. There is no source term in this equation as the bacterium is an obligate parasite. As the stochastic variables are discrete valued, it is possible to solve this equation piecewise in a sequence of time windows over which these variables are constant. Over such an interval we can rewrite equation (9) as

(10) $$\frac{\mathrm{dc}(t)}{\mathrm{dt}}=A-\mathrm{\delta c}(t),$$

in which *A* is constant. This equation has the solution

(11) $$c(t)=c(0)e^{-\mathrm{\delta t}}+\frac{A}{\delta }\left(1-e^{-\mathrm{\delta t}}\right),$$

which allows forward calculation in time of *c*(*t*) by inserting its value just prior to any change in the random variables as *c*(0), with the new value for *A* arising from the change.

### Control measures

We have examined simple control measures which consist of annual test and immediate cull of positive animals using simulated faecal culture and ELISA tests, as well as infection management, which reduces both contact between dam and calf and the excess infection arising from localized exposure to clinical animals. The calf exposure and isolation of clinical case infection management methods have been run together, without examining their effects separately. They have also been examined in combination with each of the test and cull strategies.

Reduction of the level of calf exposure from the dam was modelled by reducing the dam-calf contact parameter *ϕ* in equation (5) from 1 to 0.1, which simulates reducing the level of *post partum* infection through routes such as infected milk or colostrum, although the probability of infection *in utero* remains unchanged. The impact of management on transmission from exposure to clinical individuals was examined by reducing the contact parameter *β*_*d*_ to zero.

Note that the ELISA test, as well as being used in the simulation of control, was also used to model the collection of the reference data set for the fitting procedure described in section “Latin hypercube sampling and reweighting parameter sets”.

#### ELISA testing

An ELISA testing process was modelled in which each animal is tested independently. Based on the commercial HerdChek ELISA kit [29], it was assumed that a proportion *E*_*s*_=0.87 of infected animals will sero-convert at a sufficient level to be detected at some point in their latent period. In order to determine at what time point this threshold is reached a random variable *E*_*τ*_ is drawn from a beta distribution, *Beta*(11.89,16.07). The parameters in this distribution have been derived from [29], in which estimates are provided of the relative proportions of animals in the different shedding states which will be successfully detected. The ELISA test was used to collect the parameterisation data set [15], making it necessary to use this test model in order to model observed values from the underlying true within-herd prevalence, prior to carrying out the reweighting. A recent review compares the accuracies of ELISA and other tests for paratuberculosis [30].

#### Faecal testing

Although a piecewise constant form was used for the purposes of calculating the amount of bacteria deposited into the environment, it was necessary to explicitly model the rate of shedding when calculating a detection probability in modelling a faecal test. Thus the shedding rate for an individual animal that has undergone a fraction *t*/*T*_*i*_ of its latent period was modelled as

(12) $$r_{s}(t,T_{i})=\left\{\begin{array}{c} 0\;\text{if}\;\frac{t}{T_{i}}<f_{1},\\ \alpha_{\min}{\left(\frac{\alpha_{\max}}{\alpha_{\min}}\right)}^{\frac{1}{1-f_{1}}\left(\frac{t}{T_{i}}-f_{1}\right)}\;\text{if}\;\frac{t}{T_{i}}\geq f_{1}, \end{array}\right)$$

We assume that the probability of detection is subject to Poisson variability, with a proportion *F*_*f*_=0.00004 of an animals daily faecal production being used in the culture. We thus arrived at a detection probability

(13) $$s(t,T_{i})=1-\text{exp}(-F_{f}r_{s}(t,T_{i})).$$

The models used in [8] and [10] both used test sensitivities which monotonically increased with the severity of the infection, although in those cases a step function with fixed parameters was chosen.

### Latin hypercube sampling and reweighting parameter sets

In order to take proper account of parameter variability and uncertainty we have used a Latin hypercube sampling scheme [5], combined with a novel reweighting procedure, which selects parameter combinations that best reflect empirical data. Among the parameters in the model, some are based on fairly consistent evidence, are considered unlikely to vary greatly between farms or may be considered less critical as the model is less sensitive to them. These parameters were not used as sampled parameters, but instead assigned point estimates, and are described in Additional file 1: Table S1. While there is a degree of judgment required in the choice of these parameters and a trade-off between the number of sampled parameters and the efficiency of the procedure, other models do not account for uncertainty in parameter values, effectively choosing all parameters to be fixed, [8-11], although in most cases the sensitivity of the model to each parameter is explored. For the sampled parameters, expert opinion together with a range of published estimates has enabled the calculation of an appropriate range and distribution for their values, as described in Additional file 2: Table S2. Samples were generated from the joint distribution of these parameters, assuming independence, by Latin hypercube sampling [31,32], in which each parameter distribution is divided into equally probable intervals and combined into parameter sets using a Latin hypercube design in which each parameter interval appears once. Thus to generate *K* parameter vectors, each sampled parameter distribution is divided into *K* equally probable intervals. The generation of the input parameter sets was carried out in R [33] using the ‘lhs’ package.

As no correlation between the parameters has been assumed, the set of parameter combinations generated in this manner will contain some combinations which would have very low probability if the true joint distribution were known. Typically information on correlation between such parameters is not available. In order to generate results over the ensemble of parameter sets a prevalence data set was used to derive a set of weights, effectively picking out the parameter combinations which gave rise to results which best reflected the data. To do this a reference scenario was used in the model which recreates the conditions under which the data were collected. The best dataset identified for this purpose was that of Boelaert *et al.*[15], describing the Belgian cattle population, as no comparable data for UK cattle populations were identified. Boelaert *et al.* present this data in the form of a median and quartiles of the within-herd seroprevalence for different herd types including data for dairy cows. This necessitated the recasting of model outputs into the seroprevalences obtained by the ELISA test model, described above, in order to generate these weights using comparable data.

For a sample parameter set labeled *θ*, the equilibrium simulated true and simulated observed within-herd prevalence distributions, which we denote *p*_*θ*_(*P*) and ${\tilde{p}}_{\theta }(P_{\mathrm{obs}})$ respectively, were formed. This was done by running many realisations of the model for each parameter set *θ*, without use of any control methods other than the removal of clinical animals. Each run was left for a burn-in period of 50 years in order to ensure equilibrium had been reached before taking the prevalence, either directly or through the simulated test, and generating the corresponding prevalence distribution from the set of values obtained. Given that the seroprevalence distribution in the data is the within-herd distribution among infected farms, the distributions needed for generating weights are the *zero censored* distributions defined by

(14) $$q_{\theta }(P)=\frac{p_{\theta }(P)}{1-p_{\theta }(0)}\;P>0,$$

for the underlying prevalence, together with

(15) $${\tilde{q}}_{\theta }(P_{\mathrm{obs}})=\frac{{\tilde{p}}_{\theta }(P_{\mathrm{obs}})}{1-{\tilde{p}}_{\theta }(0)}\;P_{\mathrm{obs}}>0,$$

for the prevalence observed by ELISA test. If *p*_*θ*_(*P*) and ${\tilde{p}}_{\theta }(P_{\mathrm{obs}})$ are well defined probability distributions it is straightforward to see that *q*_*θ*_(*P*) and ${\tilde{q}}_{\theta }(P_{\mathrm{obs}})$ are also.

**Figure 1:**
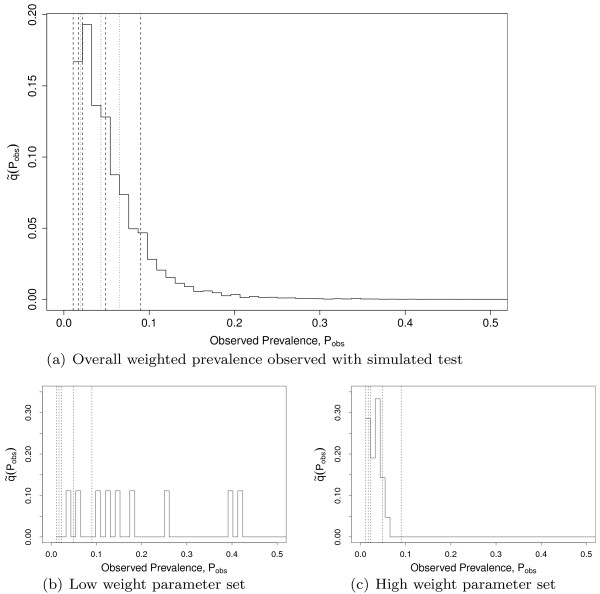
**(a) Distribution**$\tilde{q}{(P}_{\mathrm{obs})}$**of observed within-herd prevalence,*P***_***obs***_**, as observed using the simulated ELISA test and after conditioning on*P***_***obs***_***>0.*** This replicates how the prevalence data used in this paper, from [15], were collected. Also shown (dashed vertical lines) are the quartile boundaries of that data (denoted in the text as *Q*_0_to *Q*_4_), and the first, second (median) and third quartile boundaries of $\tilde{q}(P_{\mathrm{obs}})$, showing the fit of these to *Q*_1_to *Q*_3_. (b) and (c) Distribution of observed prevalence for two individual parameter sets. **(b)** is from a set of parameters which is given a low weight by the reweighting algorithm as it does not represent the data well, while **(c)** is given a higher weight.

For each parameter set the probability $q_{\theta }^{k}$ of the observed seroprevalence falling into quartile *k* of the dataset (see Figure 1) was calculated using

(16) $$q_{\theta }^{k}=\int_{Q_{k-1}}^{Q_{k}}{\tilde{q}}_{\theta }(P)\mathrm{dP.}$$

Each parameter set was assigned a weight for that quartile

(17) $$W_{\theta }^{k}=\frac{q_{\theta }^{k}}{\underset{\theta }{\sum }q_{\theta }^{k}}.$$

The final weights for each parameter set are given by combining the $W_{\theta }^{k}$ for each quartile,

(18) $$W_{\theta }=\frac{1}{4}\overset{4}{\underset{k=1}{\sum }}\frac{q_{\theta }^{k}}{\underset{\theta }{\sum }q_{\theta }^{k}}.$$

This is consistent with the concept that the observed prevalence distribution has been generated by a set of farms exhibiting between farm variability in parameter values. Parameter sets which give rise to distributions which are generated by many other parameter sets will be given lower weight, due to the normalisation factor in this equation, whereas parameter sets that generate results in quartiles for which other parameter sets infrequently produce results will be given higher weights. The overall effect of this weighting is to ensure that the weighted outputs from all the parameter sets, when run under the reference scenario, match the observed quartiles from the data.

The set of weights was generated by running the reference scenario described above, which was designed to represent the conditions under which the data were collected, but once the final weights have been generated it is possible to run alternative scenarios, such as those with control present, and hence to form weighted prevalence distributions for different control options. In all such cases the weights used should be those generated as described above using the reference scenario.

## Results and discussion

Figure 1 shows the results of carrying out the reweighting for the Belgian data set, including both the data and reweighted model quartiles, together with the model output for two individual sample parameter sets. The parameter combination used to generate Figure 1b shows a distribution with support over a wide range of within-herd prevalences. This does not reflect the data quartiles, also shown, and so generates a low weight, although as the distribution does have some support at low prevalences its weight is not zero. The variability in the outcomes from the stochastic model gives rise, for some parameter sets, to within-herd prevalences in the upper tail, which are not well supported in the data set. Many such parameter sets may still be assigned non-zero weight because other realisations with the same parameters have prevalences which are within the data quartiles, especially if these values are poorly represented by other parameter sets. This reflects the fact that the quartiles in the weighting equation (18) are treated independently. Assigning non-zero weight to distributions which have support outside of the data quartiles gives rise to an overall model prevalence distribution with a significant tail beyond the quartiles, as seen in Figure 1a. The slight over dispersion resulting from treating the quartiles independently may be beneficial in that very high prevalences, which are likely to be poorly represented in any data set compared to reality as they are low probability events, are still accounted for in the weighted results. Keeping this additional variability also addresses the need to strike a balance between the information contained in the prevalence data and that in the literature estimates used for the parameter distributions.

**Figure 2:**
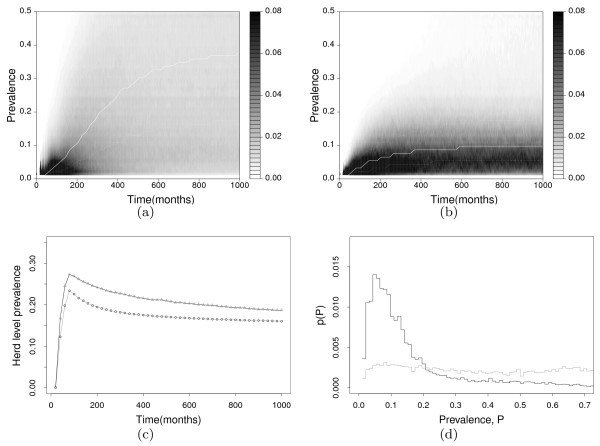
**(a) and (b) Density plots showing the distribution*q(P)*of simulated true within-herd prevalence,*P*, conditioned on*P*>0, as a function of time.** Along a vertical line (at a fixed time) the density corresponds to the height of the distribution for different prevalences (similar to Figure 1(a) but for true rather than ELISA observed prevalence). **(a)** shows the distribution before applying the reweighting algorithm, **(b)** shows the same distribution with reweighting. Also shown are the first, second (median) and third quartile boundaries of these distributions as a function of time, with the median shown by the solid white line and the outer quartiles by the dotted lines. The initial condition has no infected individuals but the environmental contamination equivalent to a single high shedding animal. It can be seen that the distributions approach equilibrium. **(c)** shows the time dependence of the corresponding herd level prevalence (i.e. the proportion of farms with infected animals) comparing the value before (circles) and after (triangles) the reweighting. **(d)** The distribution *p*(*P*) for both non-weighted (circles) and weighted models (triangles), at a single timepoint where the system is deemed to be in equilibrium.

Figure 2 shows how this procedure affects the underlying distribution of within-herd prevalence, rather than that observed through ELISA testing. It is important to note that the fitting of the variability by the weighting is not an artifact of choosing narrow parameter distributions - the choice of input parameter distributions was made to be wide enough to encompass all reasonable values, and this conservative choice is reflected in the large variability displayed before fitting in Figure 2a. The time evolution of both the zero censored distribution and herd level prevalence, 1−*p*(0), are shown in both the weighted and unweighted cases. Also shown is a snapshot of the within-herd prevalence distribution after a large time has elapsed, where the system is assumed to be at equilibrium. From Figures 1 and 2 it can be seen that using a single set of parameters is unlikely to give a good reproduction of the data set, and that it is important to capture the variability that arises from both true between farm variability in parameter values and lack of knowledge. Introducing distributions for key parameters allows for this variability, with an extremely broad distribution of within-herd prevalences evolving from the initial state (Figures 2a and 2c), but this approach still fails to reproduce typical within-farm prevalences seen in the field, giving little confidence in the predictive power of such a method without reweighting. This failure arises partly from the presence of unrealistic values for parameters, and partly from the lack of any correlational links between them. Both of these factors are mitigated by the use of the reweighting scheme, for which it can be seen that both good agreement with the data and a more appropriate level of variability is achieved (Figures 2b and 2d). Using this procedure gives a much stronger basis upon which to simulate the effect of control strategies.

**Figure 3:**
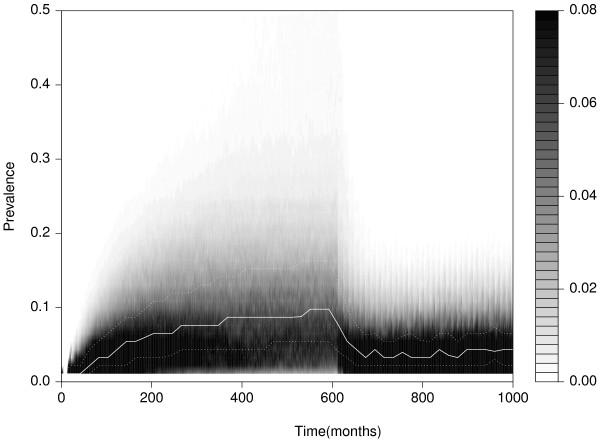
**Evolution of herd level prevalence with control.** Time evolution of the distribution *p*(*P*) of the underlying (rather than ELISA observed) prevalence, with the introduction of an ELISA test and cull strategy at *t*=600 months.

When examining control, the system was allowed to reach an equilibrium by being run for a burn in period before the control begins, after which the system was run for a further 30 years with the control strategy in place. In order to show the effect of control on the time evolution of the system, Figure 3 displays an equivalent density plot to that shown in Figure 2b, with the addition of an annual ELISA test and cull strategy commencing after the burn in period. It is clear that this strategy is highly unlikely to achieve eradication, as even after 30 years of control, there is still a significant probability (*p*=0.06 from an initial level of *p*=0.1) that a typical farm will be infected, with the rate of change of this value indicating that it is broadly at equilibrium.

**Figure 4:**
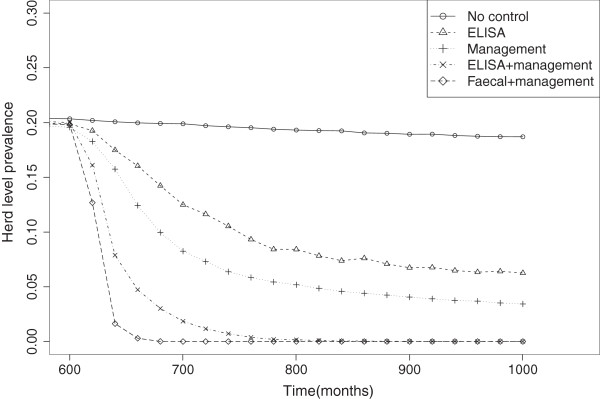
**Effect of control on herd level prevalence.** The effect of a range of control strategies on the herd level prevalence, 1−*p*(0) (i.e. the proportion of farms with infected animals) over time. The control strategies are in place throughout the period shown on the x-axis. The strategies shown are: no control (∘), annual ELISA test and cull positive animals (△), infection management by reduction of both calf exposure and infection by clinical animals ( + ), ELISA and infection management together (×) and annual faecal culture test and cull positive animals combined with infection management (◇).

Figure 4 shows how the herd level prevalence decreases with time under various control strategies. Although the combination of faecal test and cull with management was the most effective, the model used here does not incorporate a number of practical difficulties associated with using this approach in the field, such as the delay involved in carrying out the test and the cost. It is notable, however, that through this approach eradication can be achieved in 8 to 9 years. Neither management alone, nor ELISA test and cull strategies are able to eradicate the infection, however both lead to a drop in both herd level prevalence and the within-farm prevalence on infected sites, reducing the probability of a farm being infected from 0.21 to equilibrium values of 0.04 and 0.06 respectively. Finally it can be seen that combined ELISA test and cull with management leads to eradication in a time frame of around 20 years.

**Figure 5:**
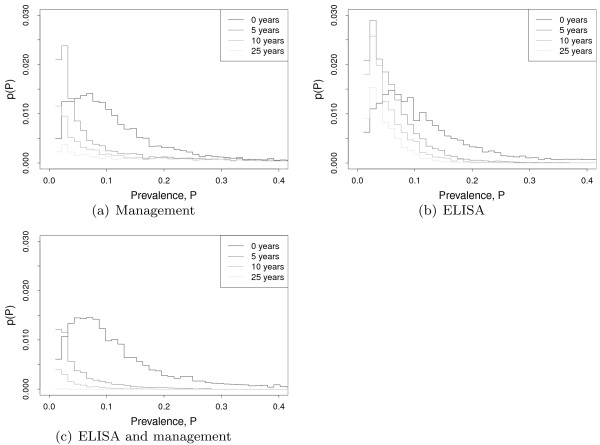
**Comparison of control options.** Distributions *p*(*P*) of the within herd prevalence *P*, conditioned on *P*>0, for three different control strategies and showing different timepoints, before the control commences and 5, 10 and 25 years after it starts. In all cases the mode of the distribution moves from higher to lower prevalence, showing that farms have fewer infected animals the longer the control strategy has been in place. **(a)** Infection management by reduction of both calf exposure and infection by clinical animals. **(b)** Annual ELISA test and cull positive animals. **(c)** ELISA and infection management together. The combination of ELISA and management can be seen to have a significant long term effect on prevalence.

The distributions of the within-herd prevalences are shown in Figure 5 for various timepoints beyond the start of control, comparing strategies with and without management and ELISA test and cull. From these figures it can be seen that the use of ELISA test and cull has a limited and slow acting impact upon low prevalence farms, reflecting the low sensitivity of the test at the onset of infection. This is a consequence of the use of the beta distribution in the ELISA model. As high shedding individuals are detected and removed from the population the effective sensitivity drops, demonstrating a fundamental limitation of any test which is not effective at the early stages of infection. By contrast, infection management predominantly targets younger animals, preventing infection from occurring and hence targeting high and low within-herd prevalence outbreaks equally. Although it is not able to completely remove infection, it is able to compensate for the shortcomings of the ELISA test and consequently the combined strategy is shown to be effective.

Control strategies have been run here for 30 years in order to observe their effect over timespans which may be of interest in practise. Over timescales of this magnitude it is likely that the values of parameters or other environmental factors will vary, and this change is not reflected in our analysis as the control options are evaluated in a fixed environment. If large scale longitudinal data were available the methods described here would be able to handle such change by using a combination of time dependent parameter distributions and weightings, thus allowing an investigation of these effects by comparison with the fixed environment examined here.

The reweighting approach implicitly allows for variation in sensitive or poorly understood parameters through the sampling algorithm. Where changes in a parameter have a strong effect on the outcome of the model, the reweighting will tend to assign an appreciable weight only to a narrow sub-range of the initial distribution. Thus, whilst the approach does not allow for the sensitivity of individual parameters to be quantified, such sensitivity is fully accounted for in the results.

## Conclusions

A practical method of accounting for uncertainty and between farm variability in realistic models of disease transmission has been developed and shown to be an effective tool for predicting their behaviour. The example of paratuberculosis in dairy herds has been explored, a disease for which there is considerable uncertainty in the values of many key quantities affecting the dynamics. Good agreement has been shown with the experimental within-herd prevalences in the control free scenario, so that with the inclusion of uncertainty and variability greater confidence can be felt in the extrapolations into the use of control.

The model developed here includes many of the key features of paratuberculosis infection, such as vertical infection as well as infection through a contaminated environment, important details when considering the common approaches to managing and eradicating infection that have been used here. Using this model in combination with the reweighting scheme, we have been able to reproduce the within-herd prevalence distribution seen in the field data. Regions with different distributions or where similar results are observed under different management or control scenarios will generate different weightings, and hence evaluations of the effectiveness of treatments will vary. This approach is able to deal with such variations and would be applicabe to making detailed regionalised predictions on the effectiveness of different control strategies. It would also be straightforward to incorporate post-control data into the generation of the weights, giving additional potential sources for increasing the robustness of the weighting scheme. Furthermore it is anticipated that it will be possible to apply the reweighted LHS technique to other diseases and other models more generally.

The use of statistical approaches to parameterising models based on field data allows greater insight into the relative strengths of different control strategies and into the range of possible outcomes and associated variabilities, particularly in cases such as paraTB in which there are a large number of biological parameters whose values are poorly understood.

## Author’s contributions

IJM and MH developed the study design, IJM conceived the reweighted LHS methodology. The underlying paraTB model was initially implemented by JCW and then subsequently by RSD, with specific advice on the design, parameterisation and various aspects of paraTB from MS, KS, AG and MH. The sampling and weighting schemes were initially implemented by JCW and further developed by RSD with advice from IJM and GM. The manuscript was prepared by RSD. All authors have read and approved the final manuscript.

## Supplementary Material

Additional file 1 **parameter_TableS1.** Model fixed parameters. Click here for file

Additional file 2 **parameter_TableS2.** Sampled parameters incorporated into the model via LHS. Click here for file
