# Supplementary material for: Accounting for uncertainty in model-based prevalence estimation: paratuberculosis control in dairy herds
Source: BMC Vet Res. 2012 Sep 10;8:159. doi: 10.1186/1746-6148-8-159 (PMC3544565; doi:10.1186/1746-6148-8-159)
Supplement: Additional file 1 — parameter_TableS1. Model fixed parameters. [file 1746-6148-8-159-S1.pdf]

## Table 1: Model fixed parameters

| Parameter | Definition | Value | Derivation |
|---|---|---|---|
| $N$ | Number of milking cattle in herd | 90 | [1] |
| $p_s$ | Probability of a calf being male (and hence immediately culled) | 0.5 | Assumption, but has been previously used, e.g. [2] |
| $p_d$ | Probability of a still or abnormal birth | 0.08 | [3] |
| $\mu_c$ | Instantaneous calf mortality, 0 to 6 months | 0.00855 per animal per month | [4] |
| $\mu_h$ | Instantaneous heifer mortality, 6 to 16 months | 0.001122 per animal per month | [3] |
| $q$ | Annual cull non-age related herd cull fraction | 0.22 | [1, 5, 6] with equation (1) and the assumption of demographic equilibrium |
| $\mu_a$ | Instantaneous standard adult mortality | $-\ln(1-0.708q)/12$ | Defined and referenced in "Herd Management" |
| $\mu_p$ | Instantaneous excess mortality due to pregnancy | $-13\ln(1-0.292q)/108$ | Defined and referenced in "Herd Management" |
| $\mu_i$ | Instantaneous excess mortality due to clinical Johne's infection | 2 per animal per month | Set assuming a mean time to cull clinical animals of two weeks, based on authors experience |
| $\phi$ | Dam-calf contact parameter, controls maximum level of exposure to dam | In the range $[0, 1]$, 1 if no exposure management in place, 0 if no contact allowed | Control parameter used to explore different levels of exposure management |
| $\lambda_c$ | Scale parameter in Gamma distribution for calf sub-clinical period | 5.08 | Derived from data in [7, 8] |
| $\nu_c$ | Shape parameter in Gamma distribution for calf sub-clinical period | 9.00 | Derived from data in [7, 8] |
| $\lambda_a$ | Scale parameter in Gamma distribution for adult sub-clinical period | 4.22 | Derived from data in [8–10] |
| $\nu_a$ | Shape parameter in Gamma distribution for adult sub-clinical period | 19.00 | Derived from data in [8–10] |
| $\lambda_v$ | Scale parameter in Gamma distribution for vaccinated animal sub-clinical period | 60.46 | Derived from data in [11] |
| $\nu_v$ | Shape parameter in Gamma distribution for vaccinated animal sub-clinical period | 3.00 | Derived from data in [11] |
| $E_s$ | Proportion of animals which eventually seroconvert, allowing detection by ELISA | 0.87 | [12] |
| $E_\tau$ | Proportion of sub-clinical period $T_i$ at which animal becomes detectable by ELISA | Drawn from Beta$(11.89, 16.07)$ | [13] |
| $F_f$ | Proportion of an animals daily faecal production used in a culture based test | 0.00004 | [14] |
| $Q_1$, $Q_2$, $Q_3$ and $Q_4$ | Quartile boundaries of the fractional seroprevalence distribution used to derive weights | 0.017, 0.022, 0.049 and 0.09 | [15] |

## References


1. Anonymous: **Economic Report on Scottish Agriculture**. Tech. rep., Scottish Executive Rural Affairs Department, Edinburgh 2000.

2. Innocent G, Morrison I, Brownlie J, Gettinby G: **A computer simulation of the transmission dynamics and the effects of duration of immunity and survival of persistently infected animals on the spread of bovine viral diarrhoea virus in dairy cattle.** *Epi. Inf.* 1997, **119**:91–100.

3. Etgen WM, Reaves PM: *Dairy Cattle Feeding and Management.* New York: Wiley, 6th edition 1978.

4. Anonymous: **Calf rearing- how to get it right and minimise losses**. Tech. rep., Milk Development Council, Cirencester 1998.

5. Castle ME, Watkins P: *Modern Milk Production.* London: Faber and Faber, 1st edition 1984.

6. Forbes D, Gayton S, McKeogh B: **Longevity - controlling culling to improve herd profitability**. Tech. rep., Milk Development Council, Cirencester 2000.

7. Rankin JD: **The experimental infection of cattle with Mycobacterium johnei III: Calves maintained in an infectious environment**. *J. Comp. Pathol.* 1961, **71**:10–15.

8. Rankin JD: **The experimental infection of cattle with Mycobacterium johnei IV: Adult cattle maintained in an infectious environment**. *J. Comp. Pathol.* 1962, **72**:113–117.

9. Rankin JD: **The experimental infection of cattle with Mycobacterium johnei II: Adult cattle inoculated intravenously**. *J. Comp. Pathol.* 1961, **71**:6–9.

10. Whitlock RH, Buergelt C: **Preclinical and clinical manifestations of paratuberculosis (including pathology)**. *Vet. Clin. N. Am.-Food A.* 1996, **12**:345–356.

11. van Schaik G, Dijkhuizen AA, Huirne RBM, Kalis CHJ, Benedictus G: **Cost-benefit analysis of vaccination against paratuberculosis in dairy cattle.** *Vet. Rec.* 1996, **139**:624–627.

12. Sweeney RW: **Transmission of paratuberculosis**. *Vet. Clin. N. Am.-Food A.* 1996, **12**:305–312.

13. Collins MT: **Diagnosis of paratuberculosis**. *Vet. Clin. N. Am.-Food A.* 1996, **12**:357–371.

14. Whitlock RH, Hutchinson LT, Sweeney RW, Spencer PA, Rosenberger AE, Van Buskirk MA: **Pattern Of Detection Of M. A. Paratuberculosis Infected Cattle In Ten Dairy Herds Cultured Every Six Months For Four Years**. In *Proceedings Of The 4th International Colloquium On Paratuberculosis, Cambridge U.K.*. Edited by Chiodini R, Collins M, Bassey E, International Association for Paratuberculosis 1995:47–53.

15. Boelaert F, Walravens K, Biront P, Vermeersch JP, Berkvens D, Godfroid J: **Prevalence of paratuberculosis (Johne's disease) in the Belgian cattle population**. *Veterinary Microbiology* 2000, **77**:269–281.
