# Supplementary material for: Accounting for uncertainty in model-based prevalence estimation: paratuberculosis control in dairy herds
Source: BMC Vet Res. 2012 Sep 10;8:159. doi: 10.1186/1746-6148-8-159 (PMC3544565; doi:10.1186/1746-6148-8-159)
Supplement: Additional file 2 — parameter_TableS2. Sampled parameters incorporated into the model via LHS. [file 1746-6148-8-159-S2.pdf]

Table 2: Sampled parameters incorporated into the model via LHS

Parameter	Definition	Distribution	Derivation
n	Maximum lactation number in cattle herd, after which all surviving animals are culled.	$U(5, 12)$	Lower limit from [1], upper limit from equation (1) together with the assumption of demographic equilibrium.
$p_{\max}$	*In utero* probability of dam to calf infection for animals in the high shedding state, with low shedding animals *in utero* rate being adjusted according to equation 4.	$N(0.27, 0.004)$	Analysis of data from [2–5]. Uncertainty around mean reduced following trial runs.
u	Extent to which *post partum* infection is present in the model. Appears in equation (5)	$U(0, 1)$	This choice explores the full support of this parameter.
α_{max}	Maximum shedding rate, as shed by a clinical animal.	e^x, where $x \sim N(13.7, 5.76)$	Mean derived from [6] and standard deviation derived from [7] by assuming that the level reported there represents a 97.5th percentile of the distribution.
δ	Decay rate for bacteria in the environment.	e^x, where $x \sim N(-4.47, 0.16)$	Analysis of the data presented in [8].[1]
c_c^{min}	Parameter in environment to calf link function, specifying bacterial population above which force of infection on calves starts to increase linearly.	e^x, where $x \sim U(\ln(10^2), \ln(10^{18}))$	See footnote[2]
c_c^{max}	Parameter in environment to calf link function, specifying bacterial population above which force of infection on calves remains constant.	$c_c^{min} + e^x$, where $x \sim U(\ln(10^2), \ln(10^{18}))$	See footnote[2]
c_a^{min}	Parameter in environment to adult cattle link function, specifying bacterial population above which force of infection on animals starts to increase linearly.	γc_c^{min}, where $\gamma \sim U(1, 100)$	See footnote[3]
c_a^{max}	Parameter in environment to adult cattle link function, specifying bacterial population above which force of infection on animals remains constant.	$c_a^{min} + u(c_c^{max} - c_c^{min})$, where $u \sim U(1, 100)$	See footnote[3]
β_d	Infection rate for adult to adult local infection (per day)	e^x, where $x \sim U(-2, 7)$	In the absence of data, chosen to give a wide range of possible scenario outcomes (appreciably wider than that which can be inferred from values in [9])
β_{ci}	Infection rate for indirect calf infection (per day)	e^x where $x \sim U(-4.6, 2.3)$	Based on data from [10] and [11][4]
β_{ai}	Infection rate for indirect adult infection (per day)	$e^{-4.6(1-u)+u\ln(\beta_{ci})}$, where $u \sim U(0, 1)$	See footnote[3]

References

1. Innocent G, Morrison I, Brownlie J, Gettinby G: **A computer simulation of the transmission dynamics and the effects of duration of immunity and survival of persistently infected animals on the spread of bovine viral diarrhoea virus in dairy cattle.** *Epi. Inf.* 1997, **119**:91–100.
2. Doyle TM: **Foetal infection in Johne's disease**. *Vet. Rec.* 1958, **70**:328.
3. Lawrence W: **Congenital infection with Mycobacterium johnei in cattle**. *Vet. Rec.* 1956, **68**:312–314.
4. Seitz SE, Heider LE, Hueston WD, Bech-Nielsen S, Rings DM, Spangler L: **Bovine fetal infection with Mycobacterium paratuberculosis**. *J. Am. Vet. Med. Assoc.* 1989, **194**:1423–1426.
5. Sweeney RW, Whitlock RH, Rosenberger AE: **Mycobacterium paratuberculosis isolated from fetuses of infected cows not manifesting signs of the disease**. *Am. J. Vet. Res.* 1992, **53**:477–480.
6. Jø rgensen JB: **An improved medium for culture of Mycobacterium paratuberculosis from bovine faeces**. *Acta Vet. Scand.* 1982, **23**:325–35.
7. Cocito C, Gilot P, Coene M, de Kesel M, Poupart P, Vannuffel P: **Paratuberculosis**. *Clin. Microbiol. Rev.* 1994, **7**(3):328–45.
8. Lovell R, Levi M, Francis J: **Studies on the survival of Johne's bacilli**. *J. Comp. Pathol.* 1944, **54**:120–129.
9. Groenendaal H, Nielen M, Jalvingh AW, Horst SH, Galligan DT, Hesselink JW: **A simulation of Johne's disease control**. *Preventive Veterinary Medicine* 2002, **54**:225–245.
10. Rankin JD: **The experimental infection of cattle with Mycobacterium johnei III: Calves maintained in an infectious environment**. *J. Comp. Pathol.* 1961, **71**:10–15.
11. Collins MT, Morgan IR: **Epidemiological model of paratuberculosis in dairy cattle**. *Prev. Vet. Med.* 1991, **11**(2):131–146.
12. Whittington RJ, Marshall DJ, Nicholls PJ, Marsh IB, Reddacliff LA: **Survival and Dormancy of Mycobacterium avium subsp. paratuberculosis in the Environment**. *Appl. Env. Microbiol.* 2004, **70**(5):2989–3004.

[1] Analysis carried out by assuming an average output of bacteria per day in line with that specified by α_{max} suggests that the log of the decay rate per day has a mean value of -4.47, with a standard error of 0.095. The uncertainty around the mean is increased to allow for uncertainty in the nuisance parameters used in the analysis and to allow for between farm variability. The decay rates presented in [12] are all much higher than this value. This difference may reflect a greater impact on bacteria from the Australian climate.

[2] There are no data to inform the choice of these parameters. These options were chosen to give a wide range of possible scenario outcomes.

[3] Chosen in the absence of data to be as broad a range as possible under the constraint that the force of infection for adult cattle is less than that for calves.

[4] Analysis of data from [10] suggests that $\beta_{ci} = 2.75$ (per month). This may be an underestimate of the true figure. The model in [11] has typical incidences in the calf population consistent with values of β_{ci} in the range 0.68 to 1000, although the most typical model used there was consistent with values of β_{ci} in the range 367 to 384 (per month). The range of the distribution was initially chosen to encompass all of these values, but the upper limit was reduced following trial runs.
